# Randomised, double-blind, placebo controlled multi-centre study to assess the efficacy, tolerability and safety of Enterosgel® in the treatment of irritable bowel syndrome with diarrhoea (IBS-D) in adults

**DOI:** 10.1186/s13063-020-4069-x

**Published:** 2020-01-30

**Authors:** Anu Kemppinen, Carol Howell, Victoria Allgar, Matthew Dodd, John Gregson, Charles Knowles, John McLaughlin, Preeti Pandya, Peter Whorwell, Elena Markaryan, Yan Yiannakou

**Affiliations:** 1 Clever Cookie Ltd, Hove, UK; 2 Enteromed Ltd, London, UK; 3 Department of Health Sciences, University of York, York, UK; 4 Department of Medical Statistics, London School of Hygiene and Tropical Medicine, London, UK; 5 Queen Mary University of London, London, UK; 6 Division of Diabetes, Endocrinology and Gastroenterology, Faculty of Biology, Medicine and Health, University of Manchester, Manchester, UK; 7 Salford Royal NHS Foundation Trust, Salford, UK; 8 The Village Practice, Thornton-Cleveleys, UK; 9 Centre for Gastrointestinal Sciences, University of Manchester, Manchester, UK; 10 County Durham and Darlington NHS Foundation Trust, University Hospital of North Durham, Durham, UK

**Keywords:** Clinical trial, Diarrhoea, Enterosgel, Intestinal adsorbent, Irritable bowel syndrome, Medical device, Placebo-controlled, Randomised controlled trial

## Abstract

**Background:**

Irritable bowel syndrome (IBS) with diarrhoea (IBS-D) is a common and chronic condition that can significantly impair quality of life. The emergence of new drugs for IBS-D has been slow and there is a need for new treatments, including drug-free treatments, which are easy to use and suitable for different patient groups. Currently available drug-free treatments include Enterosgel®, an intestinal adsorbent approved for use in IBS-D and acute diarrhoea and available over-the-counter in the UK and 30 countries worldwide. The aim of this randomised, double-blind, placebo-controlled, multi-centre study is to test the efficacy and safety of Enterosgel® compared to placebo in symptomatic treatment in IBS-D.

**Methods/design:**

We will recruit 430 participants with IBS-D from approximately 30 primary and secondary care sites in England. Participants meeting the required abdominal pain and stool consistency criteria over a 2-week screening period will be randomly allocated to receive blinded treatment (Enterosgel® or placebo) for 8 weeks. This will be followed by an 8-week open-label treatment phase with Enterosgel®. Participants will be allowed to adjust their daily dosage during both phases based on their symptoms. Participants will then return to standard care and those who responded to treatment will receive a follow-up call 8 weeks later. Co-medication with loperamide will be permitted and use recorded. The primary outcome measure is the percentage of participants defined as responders for abdominal pain and stool consistency during at least 4 weeks in the 8-week blinded phase. Secondary outcome measures include stool frequency, stool consistency, abdominal pain, bloating, urgency, adequate relief, questionnaire scores and rescue medication use. Exploratory outcomes will be assessed in subsets of participants including qualitative and quantitative data on faecal microorganisms and biomarkers and gut-related measurements from magnetic resonance imaging data.

**Discussion:**

This is the first large scale randomised controlled trial investigating Enterosgel® in IBS-D. A study design with blinded phase followed by an open-label phase was chosen to encourage participation and study completion. Demonstrating that Enterosgel® is effective and safe in IBS-D could encourage adoption by patients and healthcare professionals and foster future clinical trials assessing its use in related conditions.

**Trial registration:**

ISRCTN17149988. Prospectively registered on 14 November 2017.

## Background

Irritable bowel syndrome (IBS) is a common chronic functional bowel condition characterised by symptoms of abdominal pain and/or discomfort associated with altered bowel habits, in the absence of a structural or organic cause [1, 2]. The Rome IV criteria provide the latest diagnostic criteria for IBS [3] and its three main subtypes, i.e. IBS with diarrhoea (IBS-D), IBS with constipation (IBS-C) and mixed IBS (IBS-M). The specific cause of the disorder is not fully understood [4, 5], but among other factors may include genetic disposition, gut immune dysfunction, immune activation, gut dysbiosis, infective and dietary triggers and changes to gut permeability [6–8].

IBS is common worldwide, with an estimated prevalence globally of 11.2% [9, 10], although a more recent study based on the Rome IV criteria for diagnosis suggests a reduced estimate of around 5.0% [11]. In the UK, the prevalence is estimated to be between 10% and 20% [12]. It occurs in all age groups, including children and the elderly, although it predominantly affects adults of working age. Internationally, the overall prevalence of IBS in women is 67% higher than in men, although there are differences in the sex-specific prevalence between geographic regions [9]. The prevalence of each subtype can vary depending on the classification used. According to the World Gastroenterology Organisation, up to one-third of cases are IBS-D, up to one-third of cases are IBS-C and IBS-M accounts for up to one-third to one-half of cases [13].

IBS imposes a substantial burden on society, impacting on patients’ quality of life, work productivity and social activities, as well as on direct and indirect healthcare costs. In the UK, direct healthcare costs include an estimated eight to ten general practice (GP) visits per year [14] and associated visits for the 29% of IBS patients who are referred to secondary care specialists before returning to primary care for their long-term management [15]. Overall healthcare costs for IBS are comparable to those of other chronic diseases with a similar prevalence, such as congestive heart failure, hypertension, asthma and migraine [16].

IBS is a challenging condition to treat mainly as a result of its complex multi-factorial nature. Currently, no single universally effective approach is available for the management of IBS [13], but lifestyle or dietary changes are often implemented as the first step of management. For example, the low Fermentable, Oligo-, Di-, Mono-saccharides and Polyols (FODMAP) diet can help symptoms [12] but can be difficult to implement without support from a dietician. Probiotic use is also becoming more common and has shown to improve symptoms in patients with IBS-D [17]. Potential treatment targets include mediators such as histamine and serotonin, which are postulated to play a causative role in IBS, and bacterial products and bile acids, which also have been implicated in the generation of IBS symptoms [7, 8]. Medications for treating IBS-related symptoms include antispasmodics, psychotropic agents, bulking agents and 5-HT receptor antagonists. However, many of these drugs have proven to be inadequate for the relief of symptoms and some have safety issues [18]. Less than one-third of patients with IBS are satisfied with their current therapy, with only 45% describing their prescription drugs as “effective” [19]. Minimal understanding of the pathophysiological aspects of the condition, poorly designed studies providing unconvincing evidence, inconsistent literature on IBS treatments and high placebo response rates (30–80%) in short term studies [20–22] are all likely to contribute to the lack of effective treatments. There is, therefore, a need for well-designed clinical trials on new therapies for IBS-D.

Previous clinical studies on intestinal adsorbents have shown some improvement in the symptoms of IBS-D, but are likely to have been underpowered for many important outcome measures [23, 24]. The rationale for the use of intestinal adsorbents in the management of IBS symptoms is their ability to bind to various mediators and toxins and remove them from the gastrointestinal tract in the stools. There is also evidence from research on the intestinal adsorbent dioctahedral smectite for enhancement of the intestinal barrier function, which counteracts disruption from pro-inflammatory cytokines [25, 26]. One of the intestinal adsorbents approved for use in IBS-D and available over-the-counter in the UK is Enterosgel®, which is a drug-free treatment developed for binding toxins and other harmful substances in the gastrointestinal tract [27]. It is suitable for different patient groups, including children and the elderly. Although there has been extensive research on Enterosgel®, including two pilot studies in IBS-D reporting a normalisation of stool frequency and form and decrease in bodily pain [28, 29], and a UK based study in acute diarrhoea [30], so far none of the conducted studies have included a placebo control arm. The difficulty has been that gel-like substances with a consistency similar to commercially available Enterosgel® (for example gelatin or starch based) could potentially have effects in the gastrointestinal tract and their suitability for use as a placebo would require validation. This randomised, double-blind, placebo-controlled, multi-centre study will use an innovative approach to overcome this challenge. The aim is to test the efficacy and safety of Enterosgel® over placebo in symptomatic treatment in 430 adults with IBS-D diagnosed according to the Rome IV criteria.

## Methods/design

### Study design

**Fig. 1:**
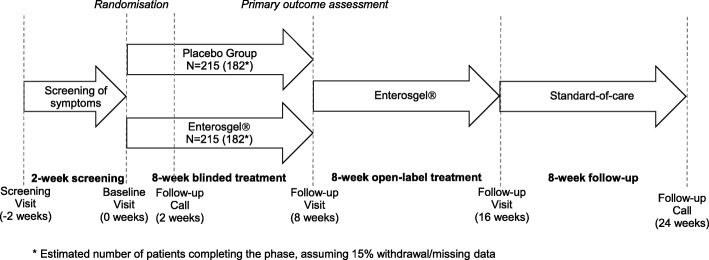
Study design

This will be a multi-centre, parallel arm, randomised, double-blind, placebo-controlled trial to evaluate the efficacy, tolerability and safety of a medical device, Enterosgel®, in the treatment of IBS-D in adults (Fig. 1). The study involves a 2-week screening phase, after which eligible participants are randomised to blinded treatment (Enterosgel® or placebo) for 8 weeks. Following the double-blind treatment phase, all participants will receive open-label Enterosgel® treatment for a further 8 weeks. At the end of the open-label treatment phase, all participants will return to standard care; however, those who responded to open-label treatment will receive a follow-up call 8 weeks later.

The study will involve four study visits and one to two follow-up calls: screening visit (− 2 weeks), baseline visit (0 weeks), follow-up call (2 weeks), follow-up visits at weeks 8 and 16 and a follow-up call at week 24 (only for participants who responded to open-label treatment).

### Eligibility criteria

#### Inclusion criteria

At screening visit, the following criteria must be met:

1. Written informed consent
2. Irritable bowel syndrome with diarrhoea (IBS-D) according to Rome IV criteria [3]
3. Aged 16–75 years
4. Considered suitable to take part in the study by the consenting investigator

At baseline visit, before starting the intervention, the following additional criteria must be met:

1. Diary completed on at least 11 of 14 days (≥ 75%) during the screening period

#### Exclusion criteria

At screening visit, the participant will not be eligible to proceed if they meet any of the following criteria:

1. Previously diagnosed coeliac disease (must be confirmed from medical records before randomisation)
2. Previously diagnosed IBD (must be confirmed from medical records before randomisation)
3. Previous bowel cancer or bowel resection (must be confirmed from medical records before randomisation)
4. Other previously known gastrointestinal disorder contributing to the diarrhoea according to principal investigator’s or sub-principal investigator’s evaluation (must be confirmed from medical records before randomisation)
5. Unexplained weight loss
6. Unexplained rectal bleeding (not including a short history of typical haemorrhoidal bleeding in patients aged < 45 years)
7. Previous use of Enterosgel®
8. Use of antidepressant agents, unless used at a stable dose for at least 6 weeks
9. Use of any probiotic supplements, other intestinal adsorbents (activated charcoal, kaoline, diosmectite), slow-release medications or strong opioids (World Health Organisation Step III) (must be confirmed from medical records before randomisation)
10. Participation in any research where treatment is provided, or was provided in the last 3 months
11. Pregnancy or not willing to use contraception for the duration of the study screening and treatment periods

At baseline visit, the participant will not be eligible to proceed to randomisation if they no longer meet one or more of the criteria above, or if they demonstrated:

1. Loose stools (Bristol Stool Form Scale (BSFS) 6 or 7) on less than 3 days during the 14 days after screening visit, and/or
2. Average abdominal pain < 3 during the 14 days after screening visit (scale 0–10: 0 = no pain; 10 = worst possible pain).

### Interventions

#### Experimental intervention

The commercially available Enterosgel® product contains 30% water and 70% polymethylsiloxane polyhydrate (PMS-PH), which is a three-dimensional crosslinked polymer of methylsiliconic acid formed by polycondensation in which hydroxyl groups form stable siloxane bonds. Since over-the-counter Enterosgel® is instructed to be taken by diluting 1–1.5 tablespoons of the product in 100–200 ml water, a water-based placebo offers an alternative to a gel-like placebo. In order to enable a water-based placebo to be used as a comparator in this trial, the blinded Enterosgel® will be provided in a pre-diluted form in 90-ml tubes containing 15 g Enterosgel® in 67.5 ml potable water. The placebo will be provided in the same 90-ml tubes, each containing a single dose of treatment. Study-specific dosage instructions for the double-blind treatment period allow participants to adjust their daily dosage based on their symptoms (Additional file 1).

For the open-label treatment phase, all participants will receive Enterosgel® in standard 15-g sachets, which are identical to those available over-the-counter in the UK. Study-specific dosage instructions also allow participants to adjust their daily dosage based on their symptoms (Additional file 2).

#### Packaging, labelling and supply

Both the placebo and Enterosgel® dilutions for double-blind treatment phase are manufactured in accordance with Good Manufacturing Practice (GMP) by Bioline Products s.r.o. (CZ), packed into identical 90-ml tubes and labelled in accordance with Annex I of the European Council Directive 93/42/EEC concerning Medical Devices. All study treatment will be stored at a Medicines and Healthcare products Regulatory Agency (MHRA)-approved warehouse facility (Wasdell Group, UK). After each randomisation, the site research team will email a request form with a unique randomisation code to a dedicated email address, which can only be accessed by Sponsor’s (Enteromed Ltd, UK) unblinded study coordinators. A coordinator will check the randomisation code against a pre-generated randomisation code list to determine whether it corresponds to placebo or Enterosgel® and will then submit a shipment request to the warehouse through a secure online portal. Supplies will be dispatched for delivery directly to the study participants within two calendar days from receiving the request (next day delivery for orders submitted before 2 pm). The supplied treatment will be sufficient to cover the entire 8-week treatment phase even if the maximum dose is taken every day.

Open-label treatment will be provided in sachets containing 15 g Enterosgel® and labelled in accordance with regulatory requirements. After a participant has been entered into the open-label phase, the site research team will send a treatment request to the Sponsor who will submit a shipment request to the warehouse as described above.

Treatment use and compliance are monitored through the daily study diary where the patients are asked to record how many doses of study treatment they used each day. If they did not take any treatment on a given day, then the electronic diary would also ask to provide a reason. These data are monitored by the study team on a weekly basis and the site teams are contacted if any issues are identified. Participants are not required to return any empty or unused tubes or sachets. Should the participant run out of study treatment during the study, they can request additional supplies through their research site.

#### Concomitant interventions

Participants will be allowed to continue to take antidepressant agents at a stable dose, provided that they had been taking a stable dose for at least 6 weeks before providing written informed consent.

Use of probiotic supplements, other intestinal adsorbents (activated charcoal, kaoline, diosmectite), slow-release medications or strong opioids will not be permitted during the study. To minimise the risk that Enterosgel® could adsorb concomitant medications in the gut, it will be recommended to leave at least 2 hours before and after taking the study treatment and taking any oral medications.

Loperamide will be provided to all study participants for use as a rescue medication during the double-blind and open-label treatment phases. Participants will be advised not to make any significant changes to their diet during the trial.

### Outcome measures

#### Primary outcome measure

The primary outcome measure is the percentage of participants defined as responders for abdominal pain *and* stool consistency during at least 4 weeks in the 8-week treatment period, where:

1. An “abdominal pain intensity weekly responder” is defined as a participant who experiences a decrease in the weekly average abdominal pain score of at least 30% compared with baseline. The weekly average abdominal pain score is derived by scoring the worst pain experienced each day and taking the average for 1 week.
  AND
2. A “stool consistency weekly responder” is defined as a participant who experiences a 50% or greater reduction in the number of days per week with at least one stool that has a consistency of BSFS type 6 or 7 compared with baseline.

A participant needs to be a responder for both abdominal pain and stool consistency in the same week to be considered a responder that week.

#### Secondary outcome measures

Secondary outcome measures for the double-blind treatment phase and open-label treatment phase are:

1. Stool frequency (mean over 8 weeks and the last 4 weeks based on a daily question in the study diary).
2. Stool consistency assessed as average number of days/week with Bristol Stool Scale type > 5 (mean over 8 weeks and the last 4 weeks based on a daily question in study diary, and percentage of responders where responder is defined as a participant with ≥ 50% reduction in this outcome compared with baseline (i.e. screening period)).
3. Abdominal pain (mean over 8 weeks and the last 4 weeks based on a daily question in the study diary, and percentage of responders where responder is defined as a participant with ≥ 30% reduction in abdominal score compared with baseline (i.e. screening period)). Abdominal pain is scored on a scale from 0 to 10, where 0 means no pain at all and 10 means the worst possible pain imaginable.
4. Bloating (mean weekly score over 8 weeks and the last 4 weeks based on a weekly question in study diary). Scale of bloating is from 0 to 6, where 0 means bloating was not bothersome at all and 6 means bloating was greatly bothersome.
5. Urgency (mean weekly score over 8 weeks and the last 4 weeks based on a weekly question in the study diary). Scale of urgency is from 0 to 6, where 0 means no urgency at all and 6 means a very great deal of urgency with bowel movements.
6. Adequate relief of global IBS symptoms (percentage of participants based on a weekly question in study diary).
7. IBS Severity Scoring System (IBS-SSS) score (weekly questionnaire)
8. IBS-related Work Productivity and Activity Impairment (WPAI:IBS; weekly questionnaire to assess percentage work time missed due to IBS, percentage impairment while working due to IBS, percentage overall work impairment due to IBS, percentage activity impairment due to IBS).
9. IBS Quality of Life (IBS-QOL) score (4-weekly questionnaire)
10. Patient Health Questionnaire 12 Somatic Symptom (PHQ-12 SS) scale (4-weekly questionnaire to assess total score and individual symptoms headache (e.), tiredness (n.) and sleep (o.))
11. Use of rescue medication, i.e. loperamide (total number of days loperamide used each week and average over 8 weeks based on a weekly question in study diary)
12. Adverse events (percentage of participants reporting serious adverse event (SAE) and adverse events (AE) possibly related to treatment and total number of SAEs and AEs reported)

Data for secondary outcome measures for the follow-up phase will be collected at week 24 follow-up call using an investigator questionnaire developed specifically for this study. Data will only be collected from participants who reported adequate relief in the last 4 weeks of the open-label treatment phase. The outcomes for the follow-up phase are:

1. Maintenance of treatment benefit (percentage of participants who report increased or maintained treatment benefit at 8 weeks)
2. Enterosgel® use (percentage of participants who report having used Enterosgel® during the follow-up period; frequency of use in these participants)
3. Loperamide use (percentage of participants who report having used less loperamide during the follow-up period than before the trial)

#### Exploratory outcome measures

1. Qualitative and quantitative data for faecal microorganisms and biomarkers will be collected at baseline and at the end of double-blind treatment period (week 8) in a subgroup of 20 participants using GI-MAP™ assay (Invivo Clinical Ltd, UK) (Additional file 3). Participants will be selected for stool testing by the randomisation program at four selected sites so that ten participants from each treatment group will be tested. Data will be compared between treatment groups at week 8. Week 8 data will also be compared to baseline in all participants. Depending on the findings, other analyses might be performed in this exploratory dataset.
2. Qualitative and quantitative data for intestinal motility, fluid volume, gas content and physiology will be collected at baseline and at 4 weeks of open-label treatment period in a subgroup of 16 participants using magnetic resonance imaging (MRI; Additional file 4). MRI data will be analysed using GIQuant image processing software (Motilent Ltd, UK). Only participants recruited to the main study from the University Hospital of North Durham and Newcastle Upon Tyne Hospitals will be invited to take part in this assessment.

### Study procedures

**Fig. 2:**
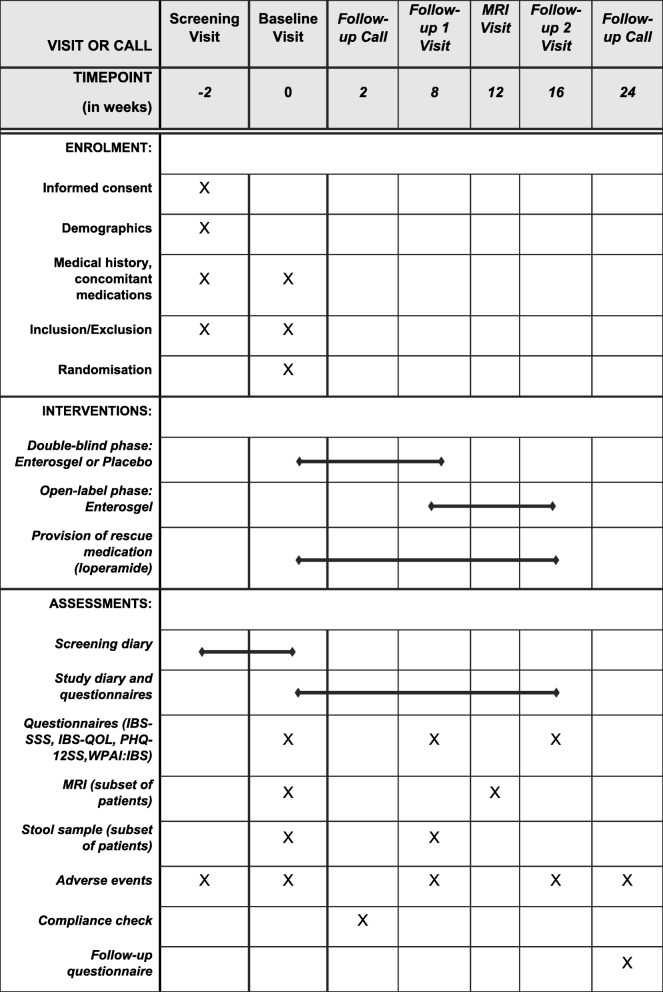
SPIRIT figure

Participants will attend four study visits and receive one to two follow-up calls from their local research team. The schedule of visits and procedures conducted at each visit are summarised in Fig. 2.

#### Screening visit (− 2 weeks)

Informed consent will be obtained before any trial-specific procedures take place. Eligibility will then be determined against all criteria except for the stool consistency and abdominal pain criteria, which will be determined over the next 2 weeks using an electronic diary or a paper diary (for participants unable or unwilling to use the electronic diary). In addition, if a participant is of childbearing potential, a pregnancy test should be conducted after the 2-week screening period if a participant is confirmed to be eligible. Any criteria related to medical history or medication use that cannot be confirmed from the participant’s medical records at screening due to these not being available to the research team, can be initially assessed based on participant-reported information. However, such eligibility criteria must be confirmed against the participant’s medical notes before the participant is randomised. Vital signs (pulse, blood pressure) should be taken and confirmed to be within the following ranges: systolic blood pressure 90–140 mmHg; diastolic blood pressure 50–90 mmHg; heart rate 50–105 beats per minute.

Demographic data and current medical conditions and concomitant medications should be recorded in the electronic case report form (eCRF).

If the participant passes all the screening steps above, they are asked to complete a daily diary for 14 days to record stool consistency and abdominal pain. Training on how to use the electronic diary will be provided. If a participant is not able or willing to use an electronic diary, they will receive a paper diary containing identical questions. The participants will be instructed not to use any antidiarrhoeal medication during the screening period.

If the participant fails screening due to lack of screening phase symptoms, they can be re-screened once if the investigator believes the level of symptoms during the initial screening period were atypical for them and that there is a likelihood of achieving symptom thresholds on a further attempt. Participants should not be informed of the detailed reason(s) for why they failed screening in order not to influence their responses if re-screened. There should be a minimum of 2 weeks from failing screening to re-screening. Participants can be enrolled into re-screening remotely with a phone call unless they need to be re-consented (i.e. if patient information has changed). Participants can also be re-screened once if their vital signs were outside accepted range at initial screening.

#### Baseline visit (week 0)

After the 14-day screening period, screening diary data will be reviewed to check eligibility against the diary-based eligibility criteria. If an electronic diary was used, the diary system will automatically evaluate eligibility. If a paper diary was used, the site investigators should enter the diary data in the electronic database to allow the system to evaluate eligibility. A negative pregnancy test must also be obtained from any female participants of childbearing potential. Participants whose eligibility is confirmed will complete study questionnaires (IBS-SSS, IBS-QOL, PHQ-12 SS, WPAI:IBS) and be randomised to blinded treatment. Participants will also receive a pack of paper questionnaires (IBS-SSS, IBS-QOL, PHQ-12 SS, WPAI:IBS) to complete at home during the treatment phase. In total, 20 study participants at selected research sites will be selected by the randomisation program for stool sample testing. A separate consent will be sought for the provision of stool samples. The selected participants who consent, will be provided with a stool sample kit and a pre-paid postage envelope to post the sample to the central laboratory where the samples will be analysed. Participants recruited to the main study from pre-selected sites will be invited to take part in MRI assessment. These participants will be provided with a separate MRI information sheet at baseline visit and will have the opportunity to discuss the assessment with the research team and ask any questions before deciding whether they wish to consent to MRI by signing a separate written informed consent form. If a participant decides not to consent to stool sample testing or MRI assessment, this will not affect their participation in the main study. Participants who consent to MRI will undergo two scans: at baseline (although not necessarily on the same day as the baseline visit) and 12 weeks later, i.e. after 4 weeks of open-label treatment. The scans will take place at Newcastle Upon Tyne Hospitals and will not last longer than 20 min involving structural and motility (cine) imaging.

#### Follow-up call (week 2)

The site research team will contact the participants to ensure that they are continuing in the study and discuss any potential issues with the diary, questionnaires or the study treatment. No data will be recorded on this call, except for any reported AEs or changes in medical history or medications.

#### Follow-up visit 1 (week 8)

Participants will complete study questionnaires (IBS-SSS, IBS-QOL, WPAI:IBS, PHQ-12 SS), and AEs and changes in medical history and medications will be reviewed. All participants will receive instructions on how to take Enterosgel® for the next 8 weeks (open label phase). Participants will also be provided with copies of the paper questionnaires for the next 8 weeks. Those participants selected for stool sample testing at baseline will receive a stool sample kit.

#### Follow-up visit 2 (week 16)

Participants will complete study questionnaires (IBS-SSS, IBS-QOL, WPAI:IBS, PHQ-12 SS), and AEs and changes in medical history and medications will be reviewed. All participants will be asked the following question (replying (a) yes or (b) no): With regard to your IBS symptoms, compared with the way you felt before you started study treatment, have you, in the past 4 weeks, had adequate relief of your IBS symptoms? Those participants who respond yes will receive a follow-up phone call in 8 weeks. Those who respond no will receive no further follow-up from the research team and will complete the study at this visit.

#### Follow-up call (week 24)

The site research team will contact those participants who had received adequate relief from the open-label treatment for a brief follow-up interview (see “Follow-up visit 2 (week 16)” section above). Any AEs and changes in medical history and concomitant medications will be recorded.

### Sample size estimation

The sample size calculation was based on demonstrating superiority for the primary outcome, i.e. response to treatment, with 90% power at 5% significance level. Assuming a response rate of 20% in the placebo group and 35% in the active treatment group, 182 participants per treatment group are required. Assuming 15% drop-out rate, in total 430 participants will need to be enrolled. The response rate of 20% in the placebo group is based on previous studies [31, 32]. The sample size was calculated using a power calculator for binary outcome superiority trial (Sealed Envelope Ltd).

### Recruitment

The study will be conducted at approximately 30 primary and secondary care sites and private gastrointestinal clinics in England. GP surgeries acting as patient identification centres will also refer patients to participating research sites. Sites will identify potential participants opportunistically and through searches of their patient databases, waiting lists, case records and referrals. Some research sites will utilise advanced software (Clinithink Ltd, UK) to identify potentially eligible patients from their clinical databases. The study can be advertised at participating sites and in public with materials approved by the Research Ethics Committee and the Health Research Authority. The study has developed a dedicated website which enables the public to check if they may be eligible and locate the contact details of their closest participating site. Finally, the study will recruit through the ContactME IBS registry [33], which contacts potentially eligible patients with details of the study. All potentially eligible patients will be provided with a patient information sheet either when visiting the GP/hospital/clinic or by post or email. Patients should be allowed at least 24 h to consider the study information before they are consented into the study.

### Randomisation

Eligible participants will be randomised by a delegated member of the site research team to a double-blind treatment group (placebo or interventional) in a 1:1 ratio. Randomisation will be performed using a computer-based online randomisation tool (Sealed Envelope Ltd, UK). The randomisation algorithm is based on the minimisation method where treatment allocation is stratified by study centre.

### Blinding

Participants randomised to the control group will receive placebo for 8 weeks. Participants randomised to the interventional group will receive Enterosgel® pre-diluted in water for 8 weeks. Both the participants and the research teams will be blinded to the treatment allocation until the end of the study.

### Unblinding

Unblinding (code-break) should only be performed during the trial in a situation where information about the participant’s trial treatment is necessary in order to provide appropriate and optimal medical care. Requests for unblinding will first be reviewed by the principal investigator (PI) or sub-PI who evaluates the information and the importance of unblinding in the given circumstances. If they decide that unblinding is necessary to ensure appropriate medical care, an unblinding request form should be submitted through the eCRF system. Unblinded treatment allocation will then be sent to the person who requested the unblinding. In case of emergency unblinding, the PI will be responsible for deciding whether the participant should continue on trial treatment. Unblinded participants should be followed up according to the study protocol until the end of the study.

### Data management

#### Data capture

Data on IBS symptoms and treatment use will be collected using a study-specific diary, which will be available as an electronic version developed by Sealed Envelope Ltd (UK). The electronic diary can be completed online by following a link provided on daily email and text message notification. For participants who are not able or willing to use the electronic diary, a paper diary will be provided. Copies of the paper diary are also provided as a back-up to participants using the electronic diary. For double-blind and open-label diaries completed on paper, the Sponsor’s research team will complete data entry into the electronic diary database. Study data recorded on any other paper source documents (e.g. questionnaires) will be transferred by the site investigators to an eCRF developed by Sealed Envelope Ltd (UK). The eCRF will be accessible via Internet browser and will be password protected to ensure that only authorised site staff and research team members can enter the system to view, add or edit data according to their permissions. Source data will be available at the site to document the existence of the study participants and will include the original documents relating to the study (demographics, medical history, medication, informed consent forms, questionnaires).

#### Analysis and archiving

After eCRF data entry is completed, all data have been monitored and raised queries have been resolved, the database will be locked. The complete exported dataset will be transferred to the statistical programmers who will complete the analyses in accordance with the Statistical Analysis Plan. All essential documents and trial data will be held by the sponsor for a minimum of 5 years after the end of the trial. Investigator site files will be archived at the participating sites for 5 years.

#### Data monitoring

The study will be conducted in accordance with the current approved protocol, International Conference of Harmonisation (ICH) Good Clinical Practice (GCP) guidance, relevant regulations and standard operating procedures. Regular monitoring will be performed in accordance with the ICH GCP and a risk-based trial monitoring plan to evaluate compliance with the protocol and accuracy in relation to source documents. In addition, data will be regularly monitored for completeness and quality using automated programmed edit checks. Any data issues are raised as queries in the eCRF system by study monitors.

An independent Data Monitoring Committee (DMC) will monitor data collected during the study for efficacy outcomes and safety. If any issues emerge, the DMC will make recommendations regarding the continuation of the study.

### Statistical analyses

Detailed methodology statistical analyses of study data will be documented in a statistical analysis plan. This trial will be reported according to the CONSORT guidelines for clinical trials.

#### Planned analyses

Analyses will be conducted following intention-to-treat (ITT) principles with outcomes analysed according to the participant’s original, randomised group irrespective of deviations based on non-compliance. The statistician will remain blind to allocation until after the trial is complete and data locked.

All participant baseline data will be summarised descriptively by trial arm. Continuous measures will be reported as means and standard deviations while the categorical data will be reported as counts and percentages.

The primary outcome measure is the percentage of participants defined as study period responders. Where necessary, multiple imputation will be used to impute missing daily abdominal pain and stool consistency scores before the required derived variables are generated (see “Missing data” section below for further details). Once obtained, the primary outcome data will be summarised descriptively and logistic regression will be used to compare the placebo and Enterosgel® groups, with odds ratio and 95% confidence interval reported.

Secondary outcome data will be summarised descriptively at different time points by trial arm. The secondary outcomes will be analysed using either analysis of covariance (ANCOVA) models (for stool consistency and abdominal pain following multiple imputation), linear mixed effects models (continuous secondary outcomes), or mixed effects logistic regression models (binary secondary outcomes). The mixed effects models will contain indicator variables for treatment group and, where appropriate, time plus a time-treatment interaction. The models will be adjusted for the baseline measure of the outcome, where available.

Significance tests will be two-sided at the 5% significance levels unless otherwise stated. All models in the analysis of the double-blind and open-label phases will be adjusted for participant’s age and gender at baseline. Analyses will be undertaken in Stata v13 or later (to be confirmed in the final report).

#### Missing data

Multiple imputation by chained equations will be used to impute missing values in daily abdominal pain and stool consistency scores before the primary outcome is derived. A sensitivity analysis will be undertaken to compare the results using multiple imputation with a complete case analysis. The complete case analysis will only use abdominal pain and stool consistency data where participants provided scores on all 7 days within a week (i.e. weeks containing at least one missing value of abdominal pain/stool consistency will be excluded).

### Quality assurance and control

The PI will be responsible for ensuring that the site is complying with the study protocol, current version of the World Medical Association Declaration of Helsinki, ICH-GCP guidelines and the applicable regulatory requirements. The PI will be responsible for ensuring that all site staff involved in the study have been appropriately trained and are qualified to conduct their delegated tasks. All medical staff involved in this study are required to have a certificate in GCP.

#### Data handling and record-keeping/archiving

All study-related paper documents (e.g. paper diaries, questionnaires, consent forms, study logs) will be filed in the study files during the study and archived at the site for 5 years after the end of the study.

#### Case report forms and source data

Data will be recorded in the eCRF from source documents defined in source data agreement with each site. All participants receive a unique study identification number (participant study ID) and no identifying data such as name, initials or date of birth will be collected in the eCRF. Source data will be available at the sites for monitoring and auditing purposes. Source data will include the original documents relating to the study, including demographics, eligibility checklists, informed consent forms and study questionnaires.

#### Record-keeping and archiving

All essential documents and trial data will be held by the sponsor for a minimum of 5 years after the end of the trial. Investigator site files should be archived at the participating sites for 5 years and should not be destroyed until authorisation to do so has been received from the sponsor.

#### Monitoring

Monitoring will be performed according to a risk-based, study-specific trial monitoring plan by monitors delegated by the sponsor. Monitoring includes checking participant eligibility criteria for all participants and confirming that data have been recorded correctly in the eCRF and any SAEs have been correctly reported and recorded.

#### Audits and inspections

All study documentation will be accessible to auditors and inspectors. All involved parties must keep the participant data strictly confidential. The Sponsor will conduct internal audits in accordance with a study audit plan.

#### Confidentiality and data protection

Access to source documents and other essential study documents will be permitted for purposes of audits and inspections. The study participants have consented to relevant sections of their medical notes and data collected during the study to be looked at by the research team, by individuals from Enteromed Ltd or contracted by Enteromed Ltd, from regulatory authorities or from the National Health Service (NHS) Trust, where it is relevant to this research. Participants have also consented to their name, home address and phone number being shared with Enteromed Ltd, and for Enteromed Ltd to provide this information to a study supplies warehouse and a courier company for the purposes of delivery of the study treatment. No identifiable data will be collected in the eCRF or will be published in any abstracts or publications resulting from the study.

#### Biological materials

Stool samples will be taken by the selected participants at home using a provided kit that includes a postage envelope for sending the sample to the central laboratory where the samples will be received within 6 days from collection and immediately stored at 4 °C upon arrival in the laboratory. Protein aliquots will be prepared within 24 h of receipt and stored at − 20 °C until testing within 3 days. All protein testing will be performed using standard enzyme-linked immunosorbent assay (ELISA) methodology. Nucleic acids will be isolated from samples within 1–2 business days after sample receipt and isolated nucleic acids will be immediately stored at − 80 °C until testing. Analysis reports will be uploaded by the laboratory onto a secure online portal accessible by the sponsor’s research team. Results will not be shared with the research sites or the study participants. The stool samples will be destroyed by the central laboratory after the samples have been analysed.

#### Safety assessments

Types of AEs associated with medical devices and applicable for this study are defined in accordance with the European Commission guidelines on medical devices [34]. AEs will be collected throughout the study from screening visit until week 24. The following information will be recorded for all AEs: medical term of the AE (SNOMED CT terminology), start date and date of resolution, seriousness, severity, study treatment action, outcome, relationship with the study treatment and expectedness. In case of a SAE related to study treatment(s) or procedures, the participant should be withdrawn from the study. Expectedness will be determined based on known side effects listed on the latest Instructions for Use for Enterosgel®. Currently listed known side effects of Enterosgel® are nausea and constipation.

#### Reporting of serious adverse events and other safety-related events

The sponsor must report all SAEs, whether initially considered to be device-related or not, immediately to the MHRA. The Research Ethics Committee should be notified of any related and unexpected SAEs within 15 days. Reports of related and unexpected SAEs in double-blind trials should be unblinded. However, local investigators should only receive information on the code-break if it is necessary for the safety of the participant.

AEs suspected to be related only to an authorised auxiliary medicinal product (i.e. loperamide), and not resulting from a possible interaction with the investigational treatment, should be reported through the Yellow Card Scheme.

## Discussion

We present a protocol and study design for a multi-centre, randomised, double-blind, placebo-controlled trial with an open-label treatment phase. The primary objective of this trial is to determine whether treatment with Enterosgel® has a positive effect on IBS symptoms in patients with IBS-D, including stool consistency and abdominal pain.

Although Enterosgel® is already available in many countries over the counter, this is the first clinical trial in IBS-D with Enterosgel® and a placebo control arm. As many patients with IBS do not get adequate relief of their symptoms with existing treatments, we wanted to design a trial where all study participants would get an opportunity to try the active treatment. We therefore included an 8-week open-label treatment phase where all participants receive Enterosgel®. This study design is likely to increase participation and study completion rates, and also allows us to evaluate the impact of Enterosgel® in participants randomised to the placebo arm for the double-blind phase.

Enterosorbents or the more recently termed “oral intestinal adsorbents” are a group of materials with sorption properties which include activated carbons, inorganic minerals and polymeric and silicon-containing resins. They have been widely used in Commonwealth of Independent States countries for decades but are less well known or utilised by healthcare professionals or the general public in the west. One of the reasons behind this disparity may be easier access in the west to pharmaceutical interventions. Enterosgel® has been used to treat a wide range of conditions from acute intestinal infections to side effects of chemo- and radiotherapy, although many of the supporting studies have intrinsic limitations with regards to methodological design and reporting. Currently, there is need for more high-level RCTs on intestinal adsorbents; this will help encourage their uptake, inform our understanding of their action and may have implications regarding their use as antibiotic alternatives and in other gastrointestinal diseases.

One of the main challenges in clinical trials in IBS is that the placebo response is typically large; placebo response rates as high as 37.5% have been reported [35]. Suggested methods to reduce placebo response include: adding a run-in phase to exclude high-responders to placebo; assessment of anxiety and depression at baseline (may be particularly important in studies of IBS); reducing the frequency of intervention and optimizing and standardizing patient–physician relationships. However, none of these strategies have clearly shown to be effective and some may not be possible to implement in all types of studies. For example, limiting patient–physician interaction might not be appropriate in a real-world setting, while prescribing low frequency therapy is not possible without also reducing the treatment effect of active treatment. We have addressed the issue of placebo response in our sample size calculation, which assumes a 20% response rate in the placebo group. The overall response rate in the total sample will be monitored throughout the trial by a DMC so that measures can be taken if the response rate is not consistent with our pre-trial assumptions.

Another challenge for IBS trials is that there are no objective outcome measures. We will use a patient-reported primary outcome measure recommended by the US Food and Drug Administration (FDA) and the European Medicines Agency (EMA) for clinical trials in IBS [36, 37]. While this outcome measure is subjective, using a standardised recommended measure will enable the results from this trial to be more easily compared to findings from other trials. As the study primary outcome measure relies on daily completion of study diaries, in collaboration with Sealed Envelope Ltd we developed an electronic online diary that the participants can easily complete on their own mobile phone, tablet or computer by simply following daily text message and/or email links, which also serve as reminders. However, a paper diary will also be available so that participants can choose the option that works best for them. The content of the diaries was carefully considered in order not to overburden participants and, as a result, some of the questions will only be completed weekly to minimise the time participants need to spend on completing the diary each day.

The primary outcome measure evaluates the impact on the key IBS features of stool consistency and abdominal pain, but IBS can also present as various other symptoms. To assess other aspects of IBS and to allow further comparison of our results to those from other studies, we have also included secondary outcome measures that are commonly used in clinical trials in IBS (e.g. IBS-SSS and IBS-QOL). In addition, we have included exploratory measures (stool analyses, MRI) to allow us to explore the effects of Enterosgel® on a physiological level.

Possibly the greatest challenge for IBS trials performed in the UK is recruitment to time and target. Despite being a common condition, it has proved surprisingly difficult to recruit effectively to trials and there are probably a number of reasons for this:

1. Although IBS is common, patients are often discharged from regular follow-up, so there is little rapport with the research team.
2. Patients are not found in one ‘place’ within the NHS service, but are dispersed.
3. Patients in secondary care who remain in follow-up often have complex illness, with severe refractory symptoms or comorbid conditions. These patients are not ideal participants for trials.
4. Patients are often reluctant to come off laxatives/loperamide and are not keen to risk being on placebo.
5. There is a likelihood that patients with chronic illness of moderate severity are less willing to undertake trial burden compared to, for example, cancer trials where the treatment may be life-saving.

To mitigate these challenges the trial was designed with the patient in mind. Extensive patient feedback was obtained on all parts of the protocol, something that is relatively unusual in commercially sponsored trials. The protocol was reviewed by the Durham BRAG (Bowel Research patient Advisory Group). One of the important outcomes of that was the need for an open label phase. As one patient put it: “*Why would I join a trial and have a chance of just being on placebo when I can go to the chemist and buy the treatment for myself*”. The group also emphasized the importance of a low visit burden (many IBS patients are of working age) and the need for rescue therapies. In a separate meeting a group of patients completed the trial diaries and questionnaires and timed themselves. This led to a reduction in the questionnaire burden and a simplification of the diaries. In a separate survey of 55 consecutive patients attending the Chief Investigator’s outpatient clinic the patients were given a small audit questionnaire asking their views on their ownership of smartphones, access to internet and acceptability electronic diaries. This encouraged the use of an electronic diary with text-based reminders. Patients being treated with the product in advance of the trial starting were asked to comment on dose-modifying instructions.

We also took advice from GPs to make sure the inclusion and exclusion criteria were pragmatic and suitable for primary care recruitment.

We will use various channels to advertise the trial locally and nationally, including using social media, have set up a dedicated study website with a list of recruiting sites and will recruit participants through a UK-based IBS registry, ContactME-IBS [33]. Some of the research sites will use an advanced software (Clinithink Ltd, UK) to identify potentially eligible patients. As IBS is a condition that in the UK is mainly treated in the primary care setting or self-managed with over-the-counter products, our study inclusion and exclusion criteria are pragmatic and we expect our study population to be representative of the real-life population of patients with IBS. If found to be effective, Enterosgel® can offer a new treatment option for IBS-D and encourage future clinical trials in other related conditions.

### Trial status

The trial started enrolment in November 2018, with the first patient consented 27 days after all required approvals for the study protocol had been received and 8 days after site activation. This has been updated to May 2020. The latest protocol version is v.3.0 dated 23 January 2019. All substantial protocol amendments have been submitted to the North East – Tyne & Wear South Research Ethics Committee, who approved the study, and to the MHRA and HRA. All non-substantial amendments have been submitted to the MHRA and HRA.

## Supplementary information

**Additional file 1.** Treatment use instructions (double-blind phase).

**Additional file 2.** Treatment use instructions (open-label phase).

**Additional file 3.** Biomarkers included in the GI-MAP™ assay.

**Additional file 4.** MRI data analysis methods.

**Additional file 5.** SPIRIT 2013 checklist: Recommended items to address in a clinical trial protocol and related documents.

## Data Availability

The datasets used and/or analysed during the current study will be available from the sponsor on reasonable request.

## Abbreviations

AE: Adverse event
BSFS: Bristol Stool Form Scale
DMC: Data Monitoring Committee
eCRF: Electronic case report form
EMA: European Medicines Agency
FDA: Food and Drug Administration
GCP: Good Clinical Practice
GP: General practice or general practitioner
IBS: Irritable bowel syndrome
IBS-D: Irritable bowel syndrome with diarrhoea
IBS-QOL: Irritable Bowel Syndrome Quality of Life instrument
IBS-SSS: Irritable Bowel Syndrome Severity Scoring System
ICH: International Conference of Harmonisation
ISRCTN: International Standard Randomised Controlled Trial Number
MHRA: Medicines and Healthcare products Regulatory Agency
MRI: Magnetic resonance imaging
NHS: National Health Service
PHQ-12 SS: PHQ-12 Somatic Symptom scale (a modified version of the PHQ-15 questionnaire with three questions on gastrointestinal symptoms excluded)
PI: Principal investigator
REC: Research ethics committee
SAE: Serious adverse event
WPAI:IBS: Work Productivity and Activity Impairment (IBS-specific WPAI questionnaire)
