# Supplementary material for: Randomised, double-blind, placebo controlled multi-centre study to assess the efficacy, tolerability and safety of Enterosgel® in the treatment of irritable bowel syndrome with diarrhoea (IBS-D) in adults
Source: Trials. 2020 Jan 30;21:122. doi: 10.1186/s13063-020-4069-x (PMC6993329; doi:10.1186/s13063-020-4069-x)
Supplement: Supplementary file 1 — Additional file 1. Treatment use instructions (double-blind phase). [file 13063_2020_4069_MOESM1_ESM.pdf]

Treatment use instructions for research participants (double-blind phase – 8 weeks)

IMPORTANT! PLEASE READ BEFORE STARTING YOUR TREATMENT

Enterosgel® is a safe, drug-free treatment with 30 years of safety history profile without any reports of serious adverse events. There is no risk of overdose. You may contact your research nurse at any time to discuss any questions, but remember that only you know the dose that works best for you.

- **Each patient with IBS is different and only you know what dose works best for your symptoms**
- It is normal that one patient will require a maximum dose while another will feel better with the minimum dose
- Your role in determining your optimal dose is essential. Do not worry about increasing or decreasing your dose to find the dose that works best for you.
- Listen to yourself and your symptoms. Feel confident in adjusting the dose and do not worry about being flexible.
- It is important to continue treatment even if you do not see any improvement after taking the maximum dose. There could be many reasons for not seeing improvement. You might have been randomised to the placebo group, or you might just need more time for the treatment to start working for you. Try to be patient and positive!
- All patients who complete this phase, will receive the active treatment (i.e. Enterosgel®) in the second, open-label phase of this study. Therefore, even if you feel that the blinded treatment does not work for you, do not give up and remember that you will get to try the active treatment soon.
- The recommendations below are only provided as guidance for you to find your individual dose. You may always adjust your dose to suit your stool consistency and bowel movements.
- You can increase treatment to a double dose (two full tubes) up to 3 times a day if your stool is loose, or reduce to a single dose (one full tube) twice a day if your stool is normal
- **It is important to know that your bowel might start changing its habits, including slowing down and not having any stool for 1-2 days.** This is completely normal. If you do not have a bowel movement for 1-2 days, increase your intake of water and continue the treatment after the next bowel movement.

WHAT IS A SINGLE DOSE AND DOUBLE DOSE?

- **SINGLE DOSE** is one full tube
- **DOUBLE DOSE** is two full tubes

HOW TO TAKE THE TREATMENT?

- **Do not eat or take any oral medication within 2 hours from taking the study treatment**
- Shake the tube well before opening
- Open tube just before use
- Mix the contents with approximately 100ml of water (half a glass).
- Make sure to use the entire contents of the tube at one time
- Do not use if package is damaged
- Keep out of the reach of children
- Store at room temperature (between +4 to +30 °C)

HOW MANY TIMES A DAY SHOULD I TAKE THE TREATMENT?

Starting the treatment
Take **A SINGLE DOSE 2 TIMES A DAY** for **5 days**.

If your symptoms improve
Continue on this dosage.

If your symptoms do not improve at all or enough
Take **A SINGLE DOSE 3 TIMES A DAY** for **5 days**.

If your symptoms still do not improve at all or enough
Take **A DOUBLE DOSE 2 TIMES A DAY** for **5 days**.

If your symptoms still do not improve at all or enough
Take **A DOUBLE DOSE 3 TIMES A DAY** for **5 days**.

If your symptoms still do not improve at all
Return to **A DOUBLE DOSE 2 TIMES A DAY** until the rest of the study phase.

NOTE: *If you are **satisfied** with the improvement on any of the above dosages, **stay on that dosage.***

NOTE: *If on any dose you do not have a bowel movement (no stool at all) for 2 days, **stop** the treatment and increase your intake of water. After the next bowel movement, **continue** with a **single dose once a day**. You can then increase step-by-step as described above if your stool is still loose and you are not satisfied with your bowel movements.*
