# Supplementary material for: Randomised, double-blind, placebo controlled multi-centre study to assess the efficacy, tolerability and safety of Enterosgel® in the treatment of irritable bowel syndrome with diarrhoea (IBS-D) in adults
Source: Trials. 2020 Jan 30;21:122. doi: 10.1186/s13063-020-4069-x (PMC6993329; doi:10.1186/s13063-020-4069-x)
Supplement: Supplementary file 3 — Additional file 3. Biomarkers included in the GI-MAP™ assay. [file 13063_2020_4069_MOESM3_ESM.pdf]

BIOMARKERS INCLUDED IN THE GI-MAP™ ASSAY

- Bacterial pathogens (Campylobacter, C. difficile Toxins A and B, Enterohemorrhagic E. coli, E. coli O157, Enteroinvasive E. coli/Shigella, Enterotoxigenic E. coli LT/ST, Shiga-like Toxin E. coli stx1 and stx2)
- Parasitic pathogens (Cryptosporidium, Entamoeba histolytica, Giardia)
- Viral pathogens (Adenovirus 40/41, Norovirus GI/II)
- H. pylori (virulence factors babA, cagA, dupA, iceA, oipA, vacA, virB, virD)
- Normal bacterial flora (Bacteroides fragilis, Bifidobacterium spp., Enterococcus spp., Escherichia spp., Lactobasillus spp., Clostridium spp., Enterobacter spp.)
- Phyla microbiota (Bacteroidetes, Firmicutes, Firmicutes:Bacteroidetes ratio)
- Additional dysbiotic/overgrowth bacteria (Bacillus spp., Enterococcus faecalis, Enterococcus faecium, Morganella spp., Pseudomonas spp., Pseudomonas aeruginosa, Staphylococcus spp., Staphylococcus aureus, Streptococcus spp.)
- Potential autoimmune triggers (Citrobacter spp., Citrobacter freundii, Klebsiella spp., Klebsiella pneumoniae, M. avium subsp. paratuberculosis, Prevotella copri, Proteus spp., Proteus mirabilis)
- Fungi/yeast (Candida spp., Candida albicans, Geotrichum spp., Microsporidium spp., Rodotorula spp.)
- Viruses (cytomegalovirus, Epstein Barr Virus)
- Parasites (Blastocystis hominis, Chilomastix mesnili, Cyclospora spp., Dientamoeba fragilis, Endolimax nana, Entamoeba coli, Pentatrichomonas hominis, Ancylostoma duodenale, Ascaris lumbricoides, Necator americanus, Trichuris trichiura, Taenia spp.)
- Intestinal health -related (Steatocrit, b-Glucuronidase, Anti-gliadin IgA, Secretory IgA, Zonulin)
- Antibiotic resistance gene phenotypes (Clarithromycin A214C, Fluoroquinolones gyrA N87K and gyrB S479N)
- Antibiotic resistance gene genotypes (b-lactamase TEM-70, VEB-1, toho-3 and PER-2, Fluoroquinolones qnrA2, Macrolides ermA and mfE, Vancomycin vanA1 and vanC)
