# Supplementary material for: Randomised, double-blind, placebo controlled multi-centre study to assess the efficacy, tolerability and safety of Enterosgel® in the treatment of irritable bowel syndrome with diarrhoea (IBS-D) in adults
Source: Trials. 2020 Jan 30;21:122. doi: 10.1186/s13063-020-4069-x (PMC6993329; doi:10.1186/s13063-020-4069-x)
Supplement: Supplementary file 4 — Additional file 4. MRI data analysis methods. [file 13063_2020_4069_MOESM4_ESM.pdf]

Motilent

# Brief Analysis Report – Relieve Study

## Background

The RELIEVE IBS-D Study aims to determine the physiological changes in the body after ingestion of Enterosgel®. Analysis is done by Motilent using the GIQuant software to quantify small bowel motility as well as semi-automated methods for morphological assessment. Structured reports are created for each subject.
The data is initially assessed to meet standard requirements to ensure clear and good quality images are used so that the delineation process has a higher probability of accuracy.

## Methods

Several imaging sequences are used to perform the quantify 1) colonic gas, 2) small bowel water and 3) small bowel motility.

### Colonic Gas

Analysis type: Colonic Gas Content
Region of interest: Large bowel
Sequence used: COR TRUFISP BH
Number of slices: 32 images +-2
*Units: cm3*

The gas deposits show up as black pockets which are delineated (ROI's). The total gas volume is calculated by summing the gas pocket volumes. The ROI's are saved and exported into a csv file and using the slice thickness the volume calculated and completed on Excel.

### Small bowel water

Analysis type: Small bowel Water Content
Region of interest: Small colon
Sequence used: AXIAL T2 HASTE BH
Number of slices: 77 images
*Units: cm3*

The water content would show up bright white to match the spinal fluid or T2 reflective of high-water content. For our analysis we segment the bright areas. The images are assessed in a DICOM viewer. The ROI's are saved and exported into a csv file and using the slice thickness the volume calculated and completed on Excel.

Motilent

## Small bowel motility

Analysis type: small bowel Motility
Region of interest: Small bowel
Sequence used: Reference and heat maps produced by GiQuant software.
GIQuant produces a motility map for each time series which can be analysed to examine motility across the small bowel.

Using the motility reference images, all of the small bowel is segmented making sure not to mark outside the bowel wall. The ROI's are saved and then opened on the 'heat map". The motility score is determined for each image by recording the figure given of the mean (using the heat map). These are then averaged on the spreadsheet.
